# Temperament and character traits in female adolescents with nonsuicidal self-injury disorder with and without comorbid borderline personality disorder

**DOI:** 10.1186/s13034-016-0142-3

**Published:** 2017-01-13

**Authors:** Taru Tschan, Claudia Peter-Ruf, Marc Schmid, Tina In-Albon

**Affiliations:** 1 Clinical Child and Adolescent Psychology, University of Koblenz-Landau, Ostbahnstraße 12, 76829 Landau, Germany; 2 Department of Child and Adolescent Psychiatry, University of Basel, 4056 Basel, Switzerland

**Keywords:** Nonsuicidal self-injury, Borderline personality disorder, Temperament, Character, Impulsivity, Go/NoGo

## Abstract

**Background:**

Temperament and character traits of adolescents with nonsuicidal self-injury disorder (NSSI) might differentiate those- with and without comorbid borderline personality disorder (BPD).

**Methods:**

Participants were 57 female adolescents with NSSI disorder without BPD (NSSI − BPD), 14 adolescents with NSSI disorder and BPD (NSSI + BPD), 32 clinical controls (CC), and 64 nonclinical controls (NC). Temperament and character traits were assessed with the Junior Temperament and Character Inventory, and impulsivity with the Barratt Impulsiveness Scale and a Go/NoGo task.

**Results:**

Adolescents with NSSI disorder scored significantly higher on novelty seeking and harm avoidance and lower on persistence, self-directedness, and cooperativeness than CC. The NSSI + BPD group scored even higher than the NSSI − BPD group on novelty seeking and harm avoidance and lower on persistence and cooperativeness (d ≥ 0.72). Adolescents with NSSI reported higher levels of impulsivity than the CC and NC group. However, this difference was not found in a Go/NoGo task.

**Conclusions:**

The results provide further evidence for a distinct diagnostic entity of NSSI disorder.

## Background

Due to the inclusion of nonsuicidal self-injury (NSSI) in the Diagnostic and Statistical Manual of Mental Disorders (5th ed.; DSM-5) [1] as a research diagnosis in section III, further studies are needed to enable a better understanding of this behavior. Independent of classification discussions, high prevalence and comorbidity rates [2–4], low quality of life [5], and increased risk of suicidality [6] highlight the importance of further research on NSSI. Special attention should be paid to adolescents, as NSSI often has its onset during this time [4, 7]. Previously, NSSI was generally assessed as one of the nine symptoms of Borderline Personality Disorder (BPD), however only a minority of adolescents with NSSI suffer from BPD [5, 8]. Several differences in the phenomenology and functions of NSSI can be found between patients with NSSI and BPD (NSSI + BPD) and patients with NSSI without BPD (NSSI − BPD). Patients with NSSI + BPD show more frequent and severe NSSI, greater diagnostic comorbidity, more severe depressive symptomatology, suicidal ideation, and emotion dysregulation than patients with NSSI − BPD [9, 10]. Regarding functions of NSSI, adolescents with NSSI + BPD endorsed higher self-punishment, anti-suicide, and anti-dissociation functions of NSSI than adolescents with NSSI − BPD [11].

Among different personality concepts, Cloninger´s [12, 13] biopsychosocial personality model seems to be able to describe healthy as well as pathological temperament and character traits, and to differentiate between patients with and without personality disorders [14, 15]. The extended model [13] includes four temperament dimensions (novelty seeking, harm avoidance, reward dependence, persistence) and three character dimensions (self-directedness, cooperativeness, self-transcendence), see Table 1. Low levels of self-directedness and cooperativeness are characteristics for personality disorders [16].

**Table 1:** *Temperament and character* dimensions

Dimension	High level	Low level
*Temperament*		
Novelty seeking	Curious, impulsive, sensation seeking	Indifferent, thoughtful, modest
Harm avoidance	Worried, pessimistic, frightened, shy	Relaxed, optimistic, fearless, confident, talkative
Reward dependence	Sensitive, warm, dependent	Cold, secluded, independent
Persistence	Hard-working, ambitious, perfectionist	Inactive, lethargic, pragmatic
*Character*		
Self-directedness	Mature, effective, responsible, determined, high self-acceptance	Immature, unreliable, indecisive, low self-acceptance
Cooperativeness	Social tolerant, empathic, helpful	Social intolerant, critical, cold, not helpful, destructive
Self-transcendence	Experienced, patient, creative, self-forgetting, connected to the universe, spiritual	Uncomprehending, proud, unimaginative, lack of humility

Patients with BPD often show a temperament profile consisting of both high harm avoidance and novelty seeking [14, 16–18]. According to Cloninger, Praybeck, Svrakic, and Wetzel [19], a personality pattern consisting of high novelty seeking and high harm avoidance represents an approach-avoidance conflict that may cause affective instability, a core feature of BPD. Studies of adolescents with NSSI − BPD are needed to investigate the link between NSSI and the described personality pattern, especially high novelty seeking and harm avoidance. Indeed, higher levels of novelty seeking were found in adolescents with NSSI compared to adolescents without NSSI [20]. Furthermore, adolescents with depressive disorder and self-harm behavior reported more harm avoidance than those without self-harm [21].

Low self-directedness is related to self-injurious behavior in adolescents [20, 21], BPD in adolescents [18] and BPD in adults [14]. Higher levels of cooperativeness were found in female adolescents with self-harm behavior (self-injuring behavior including suicidal behavior) compared to those without self-harm behavior [22], whereas adults with BPD showed lower levels of cooperativeness than adult controls [14]. Ohmann et al. [22] offer the explanation that higher cooperativeness levels in adolescents with self-harm behavior may be related to pronounced helplessness. High self-transcendence is linked to NSSI in adolescents [20] and to BPD in adults [14]. Low reward dependence is linked to internalizing symptoms like depression and anxiety [23], but no association has been found between reward dependence and NSSI [20], nor between reward dependence and self-harm behavior [21, 22]. Kaess et al. [18] found lower reward dependence in adolescents with BPD than in clinical and healthy controls. Further, persistence is linked neither to BPD [14, 18] nor to NSSI [20] or self-harm behavior.

In summary, for BPD, most studies support the personality pattern suggested by Cloninger et al. [16, 19], consisting of high novelty seeking and harm avoidance as well as low levels of self-directedness and cooperativeness [14, 18]. Adolescents with NSSI show a similar personality pattern to adolescents with BPD, however most studies have not controlled for comorbid BPD [e.g. 20, 21]. Studies using the big five model found similar personality traits related to self-injurious behavior, namely high neuroticism (comparable to harm avoidance), low agreeableness (comparable to cooperativeness), and low conscientiousness (comparable to self-directedness and persistence) [24, 25]. One part of novelty seeking, impulsivity, might explain the difficulties self-injurers have with resisting the urge to injure themselves [26]. NSSI itself is often an impulsive act, as most of the individuals with NSSI think about the act for less than five minutes before committing it [27]. Indeed, on self-report measures individuals with NSSI indicated higher impulsivity than individuals without NSSI [26, 28, 29], and patients with repetitive self-harm reported even higher impulsivity than patients with onetime self-harm behavior [30]. However, previous research has found low convergence between self-report and behavioral measures of impulsivity [for a meta-analysis see [31].

Response inhibition, one aspect of impulsivity, can be measured with a Go/NoGo task. Janis and Nock [29] compared self-reported impulsivity with experimentally assessed impulsivity in adolescents with NSSI. While participants with NSSI scored higher on self-reported impulsivity, they did not differ from the mixed clinical and nonclinical comparison groups without NSSI on behavioral measures. This result has been replicated in studies of adults with NSSI [26, 32]. The difference between self-reported and experimentally assessed impulsivity may be explained by the measurement of different impulsivity constructs. While self-report questionnaires measure general response tendencies (traits), behavioral tasks may in fact measure spontaneous reactions that are influenced by current cognitive processes [32]. Therefore, it seems important not only to investigate impulsivity with self-report measures, but also with behavioral tasks.

In summary, previous research is consistent with the notion that certain temperament traits underlie features of BPD symptoms. However, it remains unclear, if the same pattern can be found in a sample of adolescents with NSSI disorder without BPD. None of the presented studies assessed self-injuring behavior according to the *DSM*-*5* criteria [e.g. 20–22]; whereas Hefti et al. [20] investigated a school sample, Joyce et al. [21] investigated depressed adolescents with and without self-harm behavior, and Ohmann et al. [22] investigated adolescents presenting at in- and outpatient clinics. Thus, the samples were heterogeneous. To our knowledge, no study has investigated Cloninger’s temperament and character traits in adolescents with NSSI disorder with and without BPD. Cloninger’s personality traits might be especially suitable for the distinction between adolescents with and without BPD because of its dimensional structure. Therefore, the aim of the present study was to investigate impulsivity (self-report and a behavioral measures), temperament and character traits in adolescents with NSSI disorder (according to DSM-5), and differences in personality dimensions according to Cloninger et al. [13] between adolescents with NSSI with and without comorbid BPD.

We hypothesized that there are dimensional differences in temperament and character traits between four groups of adolescents. Specifically, we addressed the following research questions.

1. Do adolescents with NSSI disorder show a different personality pattern in comparison to the clinical control (CC) and the nonclinical control (NC) groups? Taking the results of previous studies into account, we hypothesized that adolescents with NSSI disorder would show higher values on novelty seeking, self-transcendence, and harm avoidance as well as lower values on self-directedness compared to the NC and the CC groups.
2. Do adolescents with NSSI + BPD show a distinct personality pattern in comparison to adolescents with NSSI − BPD? To our knowledge, no other studies exist, and therefore this analysis was exploratory.
3. Do adolescents with NSSI − BPD report more impulsivity than the NC and the CC groups? Is this difference evident in an emotional Go/NoGo task? Because of the heterogeneous results of previous studies, this analysis was also exploratory.

## Methods

### Procedure

All participants and their parents were informed about the study and gave their written consent in accordance with the Declaration of Helsinki. The local ethics committee approved the study. First, the clinical interviews were conducted and questionnaires distributed, and then the Go/NoGo task was administered.

### Measures

#### Diagnostic assessments

To examine the participants’ current or past *DSM*-*IV*-*TR* diagnoses for Axis I disorders, we conducted two structured interviews with each adolescent. The *Diagnostic Interview for Mental Disorders in Children and Adolescents (Kinder*-*DIPS)* [33] assesses the most frequent mental disorders in childhood and adolescence. Questions for substance use disorders were asked from the adult DIPS [34]. The Kinder-DIPS has good validity and reliability for Axis I disorders (child version, *kappa* = 0.48–0.88) [35]. NSSI was assessed according to the *DSM*-*5* research criteria, with questions reformulated as criteria. Interrater reliability estimates for the diagnosis of NSSI were very good (*kappa* = 0.90). Before conducting the interviews, Master’s students in clinical child psychology underwent systematic training.

Participants were administered the *Structured Clinical Interview for DSM*-*IV Axis II disorders (SCID*-*II)* [36], to assess for personality disorders. The SCID-II has been found to be suitable for use among adolescents [37]. Interrater reliability for BPD in our sample was very good (kappa = 1.00).

The *Borderline Symptom List 95 (BSL*-*95)* [38] was used as an additional instrument to measure the degree of borderline symptomatology. The items are based on the diagnostic criteria of the DSM-IV. The self-report questionnaire shows good psychometric properties [39].

The *Junior Temperament and Character Inventory (JTCI)* [40] is a self-report measure assessing the seven temperament and character traits based on Cloninger’s [13] biopsychosocial model of personality. The scales have good levels of internal consistency, with Cronbach´s α ranging from 0.79 to 0.85 [40]. The internal consistencies within the present sample ranged from α = 0.76 to 0.82.

The *Barratt Impulsiveness Scale (BIS)* [41], German version [42] is a valid and reliable self-report questionnaire to assess impulsivity with three subscales: Attentional, motor, and non-planning impulsivity. The internal consistency within the present sample was α = 0.81.

The *Youth Self Report (YSR)* [43, 44] measures a broad range of psychopathology. The problem behavior section of the YSR consists of the following primary subscales: withdrawn, somatic complaints, anxious/depressed, social problems, thought problems, attention problems, delinquent behavior, and aggressive behavior. Two second-order scales reflecting internalizing and externalizing problems and a total problems score can be calculated. Internal consistency within the present sample was α = 0.94 for the total score, α = 0.94 for the internalizing score, and α = 0.79 for the externalizing score.

The *Beck Depression Inventory*-*II (BDI*-*II)* [45] consists of 21 items and assesses depressive symptoms. The internal consistency within the present sample was α = 0.95.

### Non-emotional and emotional Go/NoGo task

Participants were instructed to press a button as fast as possible if a Go stimulus appears on the screen and to suppress reactions to NoGo stimuli. Participants had a practice run with six trials, followed by the non-emotional Go/NoGo task. Afterwards participants completed an emotional Go/NoGo task with four combinations of angry, happy, and neutral facial expressions with 12 trials for each combination. For all runs, targets occurred on 50% of the trials. The order of the four emotional runs and the trials within each run were randomized across participants.

Facial stimuli consisted of colored angry, happy, and neutral expressions from 18 individuals (9 females) taken from the NimStimFace Stimulus set [46]. Non-emotional stimuli (“+” and “×”) were presented for 200 ms and emotional stimuli for 500 ms, after a 500 ms fixation cross. The longer presentation time for emotional stimuli was due to the higher complexity of faces compared to crosses, similar to Hare et al. [47]. The inter-stimulus interval was 1.5 s, in which a reaction was still possible. Stimuli were presented with E-Prime (Psychology Software Tools, Inc., Pittsburgh, PA, USA), and omission (no reaction to Go) and commission (reaction to NoGo) errors as well as reaction times were recorded simultaneously. Omission errors indicate inattention [48], commission errors insufficient response inhibition [49], and reaction time to Go stimuli as a measure of response bias, with faster reactions indicating a response or attention bias toward the shown emotion [50].

### Data analyses

Multivariate analyses of variance (MANOVAs) were used to compare the groups (NC, CC, NSSI − BPD, NSSI + BPD) on dependent variables such as impulsivity and psychopathology. One-way between groups analyses of variance (ANOVAs) were used and effect sizes (Cohen’s *d*) calculated to further analyze significant group differences of MANOVAs. As we were interested in specific group differences, we set up orthogonal comparisons for psychopathology, personality, and self-reported impulsivity. The first comparison contrasted the NC group with the clinical groups (CC, NSSI, NSSI + BPD), the second contrasted the CC group with the two NSSI groups (NSSI − BPD and NSSI + BPD), and the third contrasted the two NSSI groups (NSSI − BPD and NSSI + BPD). Due to the small sample size, the analyses proceeded using bootstrapping with 2000 resamples. To correct for multiple testing, p values were adjusted according to the Bonferroni-Holm procedure. All analyses were performed using SPSS version 24.

For the Go/NoGo task, a similar analytic strategy was used. First, outliers (*z*-values > 3) were excluded, then the sensitivity index *d’* (*z*(Reaction rate to Go) – *z*(Reaction rate to NoGo) was calculated, as a measure of discrimination, with lower values representing an inability to distinguish between stimuli and lower performance levels [52]. To examine group differences, the non-emotional Go/NoGo task was evaluated with a one-way ANOVA, and the emotional Go/NoGo tasks were analyzed separately for emotional Go (neutral NoGo) and for neutral Go (emotional NoGo) with MANOVAs. These analyses were calculated for the sensitivity index *d’*, errors of commission and omission, as well as for the reaction time on Go trials. If the Levene test indicated that the variance homogeneity of an outcome was violated, we transformed it for the analysis (log10 or sqrt) and if indicated, Greenhouse Geisser corrected values were used. Significance levels were set at α = 0.05.

## Results

### Participants

Participants were 167 female adolescents, aged 12–19 years (*M* = 15.94, *SD* = 1.47), recruited from different inpatient child and adolescent psychiatric units in Switzerland and Germany. Participants included 57 adolescents fulfilling the *DSM*-*5* research criteria for NSSI disorder (NSSI) but not for BPD, 14 adolescents with NSSI and BPD (NSSI + BPD), 32 adolescents with a *DSM*-*IV* [51] diagnosis other than current or past NSSI (clinical controls, CC), and 64 nonclinical adolescents who had no current or past experience of mental disorders (nonclinical controls, NC). Participants were similar with respect to age, Welch’s *F* (3, 47.19) = 0.41. Regarding nationalities, most of the participants were Swiss and German, except for two Italians, one Thai and one Pole. The three most frequent mental disorders in all groups were: major depression (37.50% in CC, 70.18% in NSSI, 78.6% in NSSI + BPD), social phobia (34.38% in CC, 36.84% in NSSI, 42.9% in NSSI + BPD), and specific phobia (28.13% in CC, 19.30% in NSSI, 35.70% in NSSI + BPD). Posttraumatic stress disorder (PTSD) was a common comorbid disorder in NSSI (14.04%) and NSSI + BPD (50%), with an additional two participants from the CC group also presenting with PTSD (6.25%). Groups differed significantly regarding the diagnoses depression, *χ* ^*2*^ (2, *N* = 103) = 11.87, *p* < 0.01, and PTSD, *p* < 0.01, according to a two-sided Fisher’s exact test. There were no significant differences regarding any other *DSM*-*IV* disorders assessed with clinical interviews. Further comorbid diagnoses of the clinical groups were dysthymia, oppositional defiant disorder, attention-deficit hyperactivity disorder, conduct disorder, bulimia nervosa, anorexia nervosa, obsessive–compulsive disorder, agoraphobia, panic disorder, and generalized anxiety disorder. Groups differed significantly regarding the number of diagnoses, *F* (2, 100) = 30.37, *p* < 0.01, with patients in the NSSI + BPD group meeting significantly more diagnoses than the other groups (*M* = 5.43, *SD* = 1.83), and the NSSI − BPD group meeting significantly more diagnoses (*M* = 3.39, *SD* = 1.36) than the CC group (*M* = 2.03, *SD* = 1.00). In addition to the number of diagnoses, significant group differences emerged for psychopathology, for both internalizing and externalizing problems (according to the Youth Self Report). NSSI + BPD scored highest, followed by NSSI, CC and NC, see Table 2. Regarding borderline symptomatology, adolescents with NSSI − BPD differed significantly from adolescents with NSSI + BPD on the subscales self-destruction and hostility. Furthermore, NSSI − BPD scored above the cut off on the subscale for social isolation.

**Table 2:** Mean (standard deviations) of characteristics of non-clinical adolescents (NC), clinical controls without NSSI (CC), adolescents with NSSI disorder (NSSI), and adolescents with NSSI and BPD (NSSI + BPD), as well as ANOVA with orthogonal contrasts and effect sizes (Cohen’s d) between non-clinical and clinical groups (NC vs. rest), clinical controls and NSSI (CC vs. NSSI and NSSI + BPD), and NSSI disorder vs. Borderline personality disorder (NSSI vs. NSSI + BPD)

Characteristic	NC	CC	NSSI	NSSI + BPD	NC vs. rest	Cohen’s	CC vs.	Cohen’s	NSSI vs.	Cohen’s
	*M* (*SD*)	*M* (*SD*)	*M* (*SD*)	*M* (*SD*)		*d*	NSSI total	*d*	NSSI + BPD	*d*
YSR	(*n* = 57)	(*n* = 28)	(*n* = 47)	(*n* = 11)	*t* (139)		*t *(139)		*t* (139)	
Total	57.60 (18.70)	81.80 (21.60)	105.38 (29.97)	134.28 (22.40)	12.56**	2.22	7.04**	1.55	4.03**	1.02
YSR ext^a^	9.79 (6.56)	12.38 (6.45)	17.47 (9.15)	30.76 (7.82)	6.77**	1.43	4.58**	1.51	3.50**	1.52
YSR int	9.83 (6.46)	23.68 (9.56)	32.49 (9.53)	41.18 (8.68)	14.66**	2.76	6.22**	1.44	3.10**	0.94
BDI^b^	7.02 (7.20)	21.89 (12.68)	33.40 (12.17)	43.20 (13.29)	13.17**	2.39	4.70**	1.31	1.82*	0.81
BSL-95	(*n* = 57)	(*n* = 25)	(*n* = 38)	(*n* = 9)	*t* (125)		*t* (125)		*t* (125)	
Total^a^	47.67 (28.69)	117.31 (68.98)	182.84 (68.26)	240.55 (70.52)	11.31**	2.42	4.01**	1.38	1.46*	0.86
Dysphoria^c^	20.56 (8.98)	26.40 (10.23)	30.53 (6.72)	33.04 (6.93)	−2.27	1.13	−.546	0.67	−.110	0.38
					*t* (94.85)		*t* (34.51)		*t* (16.74)	
Self-perception^a^	3.63 (4.37)	15.13 (14.98)	28.10 (17.06)	41.85 (20.82)	11.65**	1.85	4.30**	1.14	2.12	0.79
					*t* (96.16)		*t* (34.61)		*t* (26.40)	
Affect regulation^b^	6.04 (6.33)	19.48 (11.99)	28.66 (11.29)	36.67 (7.12)	13.42**	2.59	4.54**	1.31	2.95*	0.77
Self-destruction^b^	1.46 (2.38)	9.20 (7.91)	25.66 (11.55)	34.37 (7.88)	16.28**	3.12	8.17**	2.31	2.21**	0.81
Social isolation^b^	4.09 (4.97)	12.58 (9.65)	21.87 (12.66)	29.33 (10.46)	10.38**	1.96	4.31**	1.21	1.81*	0.62
Hostility^b^	2.34 (3.14)	4.64 (4.41)	8.82 (5.92)	14.89 (5.82)	8.36**	1.58	4.69**	1.35	2.74**	1.05
Intrusions^a^	1.34 (2.11)	6.32 (6.37)	12.13 (7.50)	20.33 (12.08)	10.51**	1.77	4.58**	1.15	1.65	0.99
JTCI	(*n* = 51)	(*n* = 26)	(*n* = 46)	(*n* = 11)	*t* (130)		*t* (130)		*t* (130)	
Novelty seeking (T)^a^	47.29 (8.20)	43.00 (8.62)	48.20 (11.61)	56.00 (8.31)	0.66	0.20	3.42**	0,96	2.39**	0.72
Harm avoidance (T)	49.33 (10.18)	59.38 (8.59)	61.35 (11.10)	69.64 (8.51)	7.32**	1.47	2.34**	0,66	2.44**	0.79
Reward dependence (T)	57.06 (8.37)	52.04 (9.20)	49.96 (10.77)	45.91 (12.03)	−4.18**	0.79	−1.64	0,39	−1.24	0.37
Persistence (T)	50.22 (10.21)	53.73 (9.93)	45.09 (11.74)	35.27 (9.70)	−2.71**	0.54	−4.92**	1.31	−2.74**	0.88
Self-directedness (C)	52.22 (10.41)	43.88 (10.45)	33.22 (11.70)	26.73 (9.81)	−8.51**	1.68	−4.97**	1.32	−1.78	0.58
Cooperativeness (C)	53.75 (8.89)	56.88 (9.21)	54.93 (11.77)	46.27 (9.70)	−0.54	0.11	−2.41*	0.62	−2.56**	0.78
Self-transcendence (C)	49.43 (9.58)	53.92 (10.68)	50.02 (9.12)	50.82 (11.81)	1.15	0.21	−1.38	0.34	0.24	0.08
BIS	(*n* = 28)	(*n* = 21)	(*n* = 29)	(*n* = 8)	*t* (82)		*t* (82)		*t* (82)	
Impulsivity (BIS)	20.76 (3.15)	20.06 (3.47)	22.97 (3.94)	26.85 (2.78)	2.99**	0.77	4.70**	1.45	2.78**	1.07
Attentional	15.61 (4.01)	14.90 (3.16)	18.25 (4.10)	20.88 (1.89)	2.67**	0.72	4.24**	1.55	1.77*	0.72
Non-planning	25.52 (4.33)	24.59 (5.13)	27.47 (5.76)	34.63 (5.07)	2.72**	0.68	4.27**	1.24	3.51**	1.31
Motor	21.16 (3.96)	20.70 (3.97)	23.21 (6.90)	25.04 (4.04)	1.46	0.39	2.24*	0.70	0.89	0.29

*YSR* Youth self report (ext = externalizing, int = internalizing); *BDI* Beck Depression Inventory-II; *JTCI* Junior Temperament and Character Inventory; *BIS* Barratt Impulsiveness Scale

Bootstrapped and Bonferroni-Holm corrected p values * p < 0.05, ** p < 0.01

^a ^log transformation, ^b^ root transformation, ^c^ reciprocal transformation

### Junior Temperament and Character Inventory

As reported in Table 2, significant group differences were shown on the temperament scales novelty seeking, *F*(3, 130) = 4.32, *p* < 0.01, η^2^ = 0.09, harm avoidance, *F*(3, 130) = 18.80, *p* < 0.01, η^2^ = 0.30, reward dependence, *F*(3, 130) = 6.47, *p* < 0.01, η^2^ = 0.13, and persistence *F*(3, 130) = 9.57, *p* < 0.01, η^2^ = 0.18, as well as on the character scales self-directedness, *F*(3, 130) = 32.71, *p* < 0.01, η^2^ = 0.43, and cooperativeness, *F*(3, 130) = 2.99, *p* = 0.03, η^2^ = 0.06. There was no significant group difference regarding self-transcendence, *F*(3, 130) = 1.28, *p* = 0.28, η^2^ = 0.03. Compared to clinical controls, adolescents with NSSI scored higher on novelty seeking and harm avoidance and lower on persistence, self-directedness, and cooperativeness. The harm avoidance score was over the cut off while the other scores were within the normal range. Adolescents with NSSI + BPD showed even higher scores for novelty seeking and harm avoidance and lower scores for persistence and cooperativeness than adolescents with NSSI − BPD. Adolescents with NSSI + BPD scored above the cut off on harm avoidance and below the cut off on persistence and self-directedness.

### Barratt Impulsiveness Scale

Regarding the MANOVA for the BIS subscales, the group main effect was significant, *F*(3, 82) = 9.21, *p* < 0.01, η^2^ = 0.25. There was no significant Group x Impulsivity interaction, *F*(6, 164) = 1.36, *p* = 0.23, η^2^ = 0.05, indicating that the group differences are the same for all three subscales of the BIS. As shown in Table 2, the subsequent one-way ANOVA yielded significant group differences regarding impulsivity for the total scale, *F*(3, 130) = 9.21, *p* < 0.01, η^2^ = 0.25, as well as for the subscales attentional, *F* (3, 130) = 7.47, *p* < 0.01, η^2^ = 0.21, and non-planning impulsivity, *F*(3, 130) = 8.32, *p* < 0.01, η^2^ = 0.23, but not for the subscale motor impulsivity, *F*(3, 130) = 2.13, *p* = 0.10, η^2^ = 0.07.

### Go/NoGo-Task

Regarding the non-emotional task, there was no significant group effect for participants’ sensitivity index, *F*(3, 151) = 0.93, *p* = 0.43, commission errors, *F*(3, 151) = 0.43, *p* = 0.73, omission errors, *F*(3, 154) = 1.22, *p* = 0.31, or reaction time, *F*(3, 147) = 2.06, *p* = 0.11. The ANOVAs for the emotional task, when emotional faces were Go trials, revealed no significant main effects or interactions except for commission errors. There was a significant main effect for facial emotion, *F*(1, 148) = 29.83, *p* < 0.01, indicating a higher commission error rate for angry faces than for happy faces. Regarding omission errors, the main effect for facial emotion reached significance, *F*(1, 155) = 65.50, *p* < 0.01, indicating a higher omission error rate for angry faces than for happy faces. For reaction time (Go), the main effect for facial emotion was significant, *F*(1, 154) = 20.95, *p* < 0.01, indicating a faster reaction to happy compared to angry faces. The ANOVAs conducted for the emotional task, when neutral faces were Go trials revealed no significant effects for the sensitivity index, commission and omission error rates. For reaction time as an outcome, only one significant main effect was found: facial emotion, *F*(1, 146) = 11.94, *p* < 0.01, indicating a faster reaction to neutral faces, when happy faces served as NoGo compared to angry faces. The means and standard deviations are displayed in Table 3.

**Table 3:** Sensitivity index d’, commission and omission errors of the Go/NoGo, as well as reaction times for go trials of non-clinical adolescents (NC), clinical controls without NSSI (CC), adolescents with NSSI disorder (NSSI), and adolescents with NSSI and borderline personality disorder (NSSI + BPD)

Condition	NC	CC	NSSI	NSSI + BPD
	*M* (*SD*)	*M* (*SD*)	*M* (*SD*)	*M* (*SD*)
*d’*				
X	0.16 (1.16)	0.31 (1.07)	−0.01 (1.30)	−0.27 (1.29)
Angry Go (neutral NoGo)	0.12 (1.66)	−0.18 (1.59)	0.02 (1.38)	−0.72 (1.46)
Happy Go (neutral NoGo)	−0.04 (1.47)	0.42 (0.87)	0.08 (1.37)	−0.86 (1.50)
Neutral Go (angry NoGo)	0.05 (1.12)	0.19 (1.19)	−0.10 (1.33)	−0.40 (1.50)
Neutral Go (happy NoGo)	0.34 (1.44)	0.36 (0.82)	0.06 (1.46)	−0.62 (1.20)
*Commission*				
X	1.95 (4.55)	2.00 (5.19)	2.02 (4.57)	3.57 (7.45)
Angry Go (neutral NoGo)	15.42 (14.80)	15.42 (11.22)	18.63 (16.92)	21.15 (16.44)
Happy Go (neutral NoGo)	8.67 (11.43)	6.67 (10.24)	8.82 (11.80)	13.39 (11.46)
Neutral Go (angry NoGo)	5.83 (9.34)	4.03 (9.89)	6.37 (9.37)	4.46 (9.31)
Neutral Go (happy NoGo)	5.42 (10.88)	3.23 (6.43)	5.19 (9.31)	6.25 (9.49)
*Omission*				
X	14.34 (13.24)	12.26 (13.09)	17.21 (15.13)	18.57 (10.46)
Angry Go (neutral NoGo)	7.38 (12.37)	10.48 (12.95)	6.37 (6.76)	11.61 (10.36)
Happy Go (neutral NoGo)	0.82 (3.12)	0.00 (0.00)	0.47 (2.40)	1.79 (4.54)
Neutral Go (angry NoGo)	2.29 (6.71)	2.92 (5.38)	3.54 (9.61)	8.65 (9.39)
Neutral Go (happy NoGo)	4.30 (16.44)	6.05 (18.78)	6.60 (18.61)	12.50 (18.99)
*RT Go*				
X	373.62 (42.10)	378.22 (41.96)	361.03 (40.66)	353.66 (29.87)
Angry Go (neutral NoGo)	514.52 (86.87)	529.93 (109.17)	509.37 (83.11)	421.31 (119.90)
Happy Go (neutral NoGo)	483.46 (72.24)	492.22 (81.30)	478.21 (78.84)	487.61 (96.52)
Neutral Go (angry NoGo)	503.67 (86.93)	522.27 (89.08)	516.01 (82.00)	517.93 (100.72)
Neutral Go (happy NoGo)	533.06 (87.16)	546.78 (106.83)	527.60 (95.38)	551.99 (89.60)

*d’* sensitivity index; *Commission* Commission error; *Omission* Omission error; *RT Go* reaction time for the go condition

There were no significant group effects

## Discussion

The aim of the present study was to investigate temperament and character traits on the basis of Cloninger's [12, 13] personality model, with a special focus on impulsivity in adolescents with NSSI disorder without BPD (NSSI − BPD), adolescents with NSSI disorder and BPD (NSSI + BPD), a clinical control group, and a nonclinical control group. As expected, the groups showed distinct personality profiles. The JTCI scales as well as most YSR scales indicate a staircase-like appearance ranging from nonclinical adolescents to adolescents with NSSI + BPD. Adolescents with NSSI disorder without BPD scored higher on novelty seeking and harm avoidance and lower on self-directedness, persistence and cooperativeness than clinical controls. In adolescents with NSSI + BPD this personality pattern was even more pronounced than in adolescents with NSSI − BPD. Thus, we were able to replicate the personality pattern consisting of high harm avoidance and novelty seeking in adolescents with NSSI + BPD, similar to Cloninger [16] and Kaess et al. [18]. The approach-avoidance conflict generated from this pattern might be a reason for the emotional instability patients with BPD experience [19]. In addition, we extended these findings to adolescents with NSSI disorder without BPD. In these patients, the personality pattern described above was less pronounced. Nevertheless, the harm avoidance score above cut off indicates that adolescents with NSSI − BPD are more careful, fearful, insecure, and negativistic than the adolescents from the CC and the NC groups. Adolescents with NSSI − BPD differed from adolescents with NSSI + BPD regarding psychopathology and partially in borderline symptomatology but nevertheless showed a similar personality pattern to adolescents with NSSI + BPD. This result underlines the need for a dimensional personality assessment to better understand adolescents with NSSI − BPD. Further research should focus on maladaptive personality traits that do not constitute a formal personality disorder and on the validation of the dimensional personality model suggested in section III of the *DSM*-*5*.

Results of the present study replicated a profile of lower levels of self-directedness in adolescents with NSSI (−BPD and +BPD) than adolescents without NSSI, similar to Hefti et al. [20] and Joyce et al. [21]. In contrast to Ohmann et al. [22], we found lower levels of cooperativeness in adolescents with NSSI compared to adolescents without NSSI, however this result is similar to the low level of cooperativeness found in adolescents with BPD [53]. Lower cooperativeness may cause more interpersonal conflict and distress through socially intolerant, critical, and destructive conflict behavior. In fact, previous research indicates that adolescents with NSSI frequently report problems in social interactions [54] that can trigger NSSI [55]. Compared to the CC group, the level of persistence in adolescents with NSSI was low but still in the normal range. Previous studies have shown that adolescents with NSSI give up faster when pursuing goals, while adolescents without NSSI are more diligent and persevering [40]. All groups were similar regarding self-transcendence, therefore, we could not find supporting evidence for a higher self-transcendence as previously reported in adolescents with NSSI [20] and adults with BPD [14]. This may be explained by differences in the study populations (school sample vs. clinical sample, female vs. male adolescents, adolescents vs. adults and NSSI vs. BPD).

To summarize, there was a significant difference in temperament and character traits between adolescents with NSSI + BPD and adolescents with NSSI − BPD, despite the small NSSI + BPD sample size (*n* = 14). Compared to the other groups, the NSSI − BPD group displayed higher standard deviations on the subscales of the JTCI, indicating the heterogeneity of this group. Considerable diagnostic heterogeneity among adolescents with NSSI has been described in earlier studies [2].

Adolescents with NSSI disorder (−BPD and +BPD) showed more novelty seeking than the CC group as well as higher scores on all subscales of the Barratt Impulsiveness Scale (attentional, non-planning, and motor impulsivity). However, this difference was not evident in the Go/NoGo task with neither a group effect, nor an emotion effect emerging. Happy faces were associated with faster reactions and a lower error rate compared to angry faces, indicating that happy faces are easier to discern than angry faces. Our results are in line with several other studies that indicated more self-reported impulsivity in adolescents [26, 29] and adults with NSSI [32], but failed to show this difference on behavioral measures. This leaves the question open, as to whether adolescents with NSSI perceive themselves as more impulsive than they actually are. However, this discrepancy between self-report and behavioral measures is not only observed in adolescents with NSSI, but also represents a general difficulty in the measurement of impulsivity that may be explained by the measurement of different impulsivity constructs [32]. It remains to be investigated, if the difference between self-reported and experimentally assessed impulsivity can be explained by the measurement of different impulsivity constructs, or if adolescents with NSSI are able to suppress their impulsivity for an experimental task. Adolescents with NSSI + BPD reported even more impulsivity than adolescents with NSSI − BPD, especially more non-planning impulsivity (lack of future orientation and foresight). Highly impulsive individuals may be especially motivated to act rashly in the context of negative emotions because long-term benefits become less important compared to short-term gains of emotion regulation, e.g. The Theory of Urgency [56], also see [57]. Therefore, individuals with high levels of non-planning impulsivity may be highly motivated to obtain the immediate benefits of NSSI (e.g., relief of negative emotions) with less concern for the long-term consequences of NSSI. There was no significant difference between adolescents with NSSI + BPD and with NSSI − BPD in the Go/NoGo task.

The results of the present study should be interpreted in the context of some limitations. The design of the study was cross-sectional. Therefore, the current study cannot explain whether certain temperament and character traits might favor the development of NSSI. This should be investigated in future prospective longitudinal studies. Nevertheless, results indicate an association between temperament and character traits and NSSI disorder. Due to the small sample sizes of adolescents with BPD, comorbidity with other personality disorders could not be included in the analyses. The recommendation of the *DSM*-*5* is to apply a diagnosis of a personality disorder in children and adolescents when maladaptive personality traits appear to be pervasive, persistent, unlikely to be limited to a particular developmental stage or another mental disorder, and after one year of persistent symptoms. Given the mean age of the participants under 16 years of age, we were careful applying a diagnosis of a personality disorder. However, despite the small NSSI + BPD sample size, significant differences emerged between adolescents with NSSI + BPD and adolescents with NSSI − BPD. The high prevalence of NSSI in inpatient samples (50%) [9] represented a challenge for the recruitment of a clinical inpatient sample without NSSI. Our sample consisted of female adolescents admitted to a psychiatric unit and therefore generalizations to male outpatients must be made with caution. Regarding the Go/NoGo task, the low error rate indicates that the response pressure was too low. Therefore, future studies should use a higher ratio of Go stimuli to NoGo stimuli.

A strength of this study was the use of the *DSM*-*5* diagnostic criteria for NSSI disorder in a clinical sample. In addition, a clinical control group of adolescents with other mental disorders without NSSI was included. This allowed us to identify temperament and character traits specific to NSSI disorder with and without BPD. To our knowledge, this is the first study comparing temperament and character traits in adolescents with NSSI + BPD and adolescents with NSSI − BPD in an inpatient setting. In addition to self-report measures, impulsivity was assessed using an experimental task.

## Conclusions

Given the differences in temperament and character traits between adolescents with NSSI + BPD and adolescents with NSSI − BPD, a personality assessment using the JTCI [40] might be useful for the diagnostic distinction between adolescents with NSSI with and without BPD. A clear distinction of these two groups might be helpful when choosing a specific treatment for adolescents engaging in NSSI. As specific treatment programs for adolescents with NSSI are still in development, practitioners mostly use treatment programs for BPD [58]. The development of specific treatment programs for adolescents with NSSI may not only optimize treatment, but also allow an early intervention, preventing chronic conditions [59]. Future studies should investigate temperament and character traits of adolescents with NSSI in the long-term as well as the effects of psychotherapy on character and temperament development.

## Abbreviations

NSSI: nonsuicidal self-injury
BPD: Borderline personality disorder
NSSI − BPD: adolescents with NSSI disorder without BPD
NSSI + BPD: adolescents with NSSI disorder and BPD
CC: clinical controls
NC: nonclinical controls
DSM-5: Diagnostic and Statistical Manual of Mental Disorders, 5th ed
PTSD: posttraumatic stress disorder
Kinder-DIPS: Diagnostic Interview for Mental Disorders in Children and Adolescents
SCID-II: Structured Clinical Interview for DSM-IV Axis II disorders
BSL-95: Borderline Symptom List 95
JTCI: Junior Temperament and Character Inventory
BIS: Barratt Impulsiveness Scale
YSR: Youth Self Report
BDI-II: Beck Depression Inventory-II
ANOVA: analyses of variance
MANOVA: multivariate analyses of variance
